# Choline and betaine intakes during pregnancy in relation to risk of gestational diabetes mellitus among Chinese women

**DOI:** 10.1017/S0007114524001995

**Published:** 2024-10-14

**Authors:** Kallie Lamkin, Lan Xu, Kaipeng Wang, Yuhong Liu, Kefeng Yang, Hui Wu, Lingpeng Lu, Xiaoxi Shen, Cassandra M. Johnson, Jie Jia, Jie Zhu

**Affiliations:** 1 Nutrition and Foods Program, School of Family and Consumer Sciences, Texas State University, San Marcos, TX 78666, USA; 2 Department of Epidemiology, School of Medicine, Shanghai Jiao Tong University, Shanghai 200025, People’s Republic of China; 3 Graduate School of Social Work, University of Denver, Denver, CO 80208, USA; 4 Department of Gynecology and Obstetrics, Shanghai Seventh People’s Hospital of Shanghai University of Traditional Chinese Medicine, Shanghai 200137, People’s Republic of China; 5 Department of Clinical Nutrition, College of Heath Science and Technology, School of Medicine, Shanghai Jiao Tong University, Shanghai 200025, People’s Republic of China; 6 Shanghai Key Laboratory of Pediatric Gastroenterology and Nutrition, Xinhua Hospital, School of Medicine, Shanghai Jiao Tong University, Shanghai 200092, People’s Republic of China; 7 Department of Clinical Nutrition, Shanghai Seventh People’s Hospital of Shanghai University of Traditional Chinese Medicine, Shanghai 200137, People’s Republic of China; 8 Department of Clinical Laboratory, Shanghai Seventh People’s Hospital of Shanghai University of Traditional Chinese Medicine, Shanghai 200137, People’s Republic of China; 9 Department of Mathematics, Texas State University, San Marcos, TX 78666, USA

**Keywords:** Choline, Betaine, Gestational diabetes mellitus, Diet intake

## Abstract

Previous animal studies found beneficial effects of choline and betaine on maternal glucose metabolism during pregnancy, but few human studies explored the association between choline or betaine intake and incident gestational diabetes mellitus (GDM). We aimed to explore the correlation of dietary choline or betaine intake with GDM risk among Chinese pregnant women. A total of 168 pregnant women with GDM cases and 375 healthy controls were enrolled at the Seventh People’s Hospital in Shanghai during their GDM screening at 24–28 gestational weeks. A validated semi-quantitative FFQ was used to estimate choline and betaine consumption through face-to-face interviews. An unconditional logistic regression model was adopted to examine OR and 95 % CI. Compared with the controls, those women with GDM incidence were likely to have higher pre-pregnancy BMI, be older, have more parities and have higher plasma TAG and lower plasma HDL-cholesterol. No significant correlation was observed between the consumption of choline or betaine and incident GDM (adjusted OR (95 % CI), 0·77 (0·41, 1·43) for choline; 0·80 (0·42, 1·52) for betaine). However, there was a significant interaction between betaine intake and parity on the risk of GDM (*P*_for interaction_ = 0·01). Among those women with no parity history, there was a significantly inverse correlation between betaine intake and GDM risk (adjusted OR (95 % CI), 0·25 (0·06, 0·81)). These findings indicated that higher dietary betaine intake during pregnancy might be considered a protective factor for GDM among Chinese women with no parity history.

Gestational diabetes mellitus (GDM) is often diagnosed in the second or third trimester of pregnancy, which was not clearly overt before gestation^(1)^. GDM affects approximately 16·9 % of pregnancies globally^(2)^ with its prevalence ranging from 5 to 24 % in China^(3)^. GDM can lead to adverse perinatal outcomes including macrosomia, caesarean delivery and shoulder dystocia^(4,5)^. In addition, GDM has been linked to long-term adverse outcomes in both mothers (e.g. higher incident type 2 diabetes later in life) and their offspring (e.g. increasing rate of adiposity and diabetes in teenagerhood or young adulthood)^(6–9)^. Therefore, identifying modifiable risk factors for GDM prevention is imperative. Despite the unclear pathogenesis of GDM, a growing body of evidence indicates that healthy diet and lifestyle factors could benefit the establishment of prevention strategies for GDM^(4,6,10,11)^.

Choline, an essential nutrient, serves as a precursor to synthesise the neurotransmitter acetylcholine^(12)^. It also contributes to the component of cell membrane and lipoprotein cholesterol^(12)^. In addition, choline can also irreversibly convert to betaine, which donates its one-carbon group for remethylating homocysteine to methionine and regulates methylation modification^(12)^. Both choline and betaine can be synthesised endogenously, but their de novo synthesis may not be sufficient^(12,13)^. Thus, exogenous sources of choline and betaine by diet are essential for optimal human health^(12,13)^.

Previous experimental studies demonstrated that choline or betaine supplementation improved glucose intolerance and insulin resistance in murine models^(14–16)^. However, human studies on choline or betaine status and GDM were scanty, and results remained inconsistent. Previous prospective cohort studies reported that circulating betaine was inversely associated with GDM risk in Chinese women^(17,18)^, whereas no significant difference in blood choline or betaine was observed between GDM and non-GDM Mexican women^(19)^ or Canadian women^(20)^. In addition, both intake and plasma levels of choline, but not betaine, were observed higher in the US pregnant women with GDM than those with non-GDM^(21)^. Moreover, it is unclear whether higher intakes of these two nutrients during pregnancy are related to lower GDM risk. Therefore, the current study aims to investigate the correlation between maternal intake of choline or betaine during pregnancy and incident GDM among a hospital-based Chinese cohort.

## Materials and methods

### Study design and population

This hospital-based case–control study included pregnant women who were recruited at 24–28 gestational weeks when they attended a universal screening for GDM at the Seventh People’s Hospital affiliated to the Shanghai University of Chinese Medicine from December 2019 to May 2021. They were followed up through the postpartum period. This study was conducted according to the guidelines laid down in the Declaration of Helsinki, and all procedures involving human subjects/patients were approved by the Medical Ethics Committee of the Shanghai Seventh People’s Hospital, Shanghai, China. Written informed consent was obtained from all study participants. This study was registered at www.chictr.org.cn (ChiCTR1900027764).

To estimate the sample size, we assumed that the type I error rate of α was 0·05, the power of test was 80 % (*β* = 20 %) and a case-to-control ratio was 1:2. Based on the previous similar study conducted among the pregnant women in Shanghai^(12)^, the proportion of choline intake in the lowest quintile in the control group was estimated as 24·8%, and considering the expected proportion of that in the case group equal to 37·8 % based on the expert opinion, the minimal required sample size was estimated as 159 in the case group and 318 in the control group. In addition, considering the response rate was 90 %, we assumed the required sample size was 530. Open Source Statistics for Public Health (OpenEpi) version 3.01 was adopted to determine the sample size^(22)^.

Eligible women for this study were those (1) who were aged 18–50 years at enrolment; (2) who were at 24–28 gestational weeks when visiting for GDM screening; (3) who were able to read, understand and sign informed consent; (4) who were either a Shanghai native or had been a resident in Shanghai for at least 5 years; and (5) who were Han Chinese with non-smoking status. The recruitment was conducted during the daytime (from 07.00 to 17.00). When one GDM woman was recruited in the case group, two healthy pregnant women were selected in the control group for each GDM case based on age (±3 years) and gestational age when the blood sample was collected (±1 week).

We originally recruited 570 pregnant women during their clinical examination at 24–28 weeks of gestation. Participants were excluded if (1) they were diagnosed with type 1 or type 2 diabetes prior to the present pregnancy (*n* 5); (2) they were taking drugs that affect choline and betaine metabolism or glucose metabolism (*n* 7); (3) implausible energy intake (≤ 600 or ≥ 4500 kcal) (*n* 4); and (4) they had severe heart, brain, liver or kidney diseases, gestational thyroid disease, placenta previa, any infectious diseases, any autoimmune diseases or any history of chemotherapy (*n* 11). In total, 543 participants (168 GDM cases and 375 controls) were enrolled in the present study.

### Ascertainment of gestational diabetes mellitus

After overnight fasting for ≥ 8 h, all enrolled pregnant women first had fasting blood drawn and then underwent a 75-g oral glucose tolerance test, which was followed by blood drawn at 1 h and 2 h post-oral glucose tolerance test. GDM was diagnosed according to the recommendation by the International Association of Diabetes and Pregnancy Study Groups if at least one of the following criteria was met: fasting plasma glucose ≥ 5·1 mmol/l, 1-h plasma glucose ≥ 10·0 mmol/l, or 2-h plasma glucose ≥ 8·5 mmol/l. The case group included women diagnosed with GDM, and the control group included healthy women with a complication-free pregnancy^(23)^.

### Dietary assessment

Dietary intake data during pregnancy (24–28 weeks of gestation) were collected using a validated semi-quantitative FFQ, which included 120 most common food items in the Chinese diet^(12)^. The dietary assessment using this semi-quantitative FFQ was administered during face-to-face interviews at the same clinical examination (24–28 weeks of gestation) by the trained registered dietitians. Serving size of food items was reported by participants using a picture book, which displayed common Chinese food items with various kitchen utensils portion sizes. Then, the consumed serving size for each food item was converted to the amount (g) adopting the conversion coefficients for each item. Food intake frequencies were recorded in times per d, week, month or never. Supplement consumption was also recorded.

Participants’ common macro- and micro-nutrient consumption was computed via entering the amounts into Nutrition Star Nutrient Calculator Software (Shanghai Zhending Health Technology Co. Ltd). This software was developed according to the Chinese Food Composition Table^(24)^. In addition, choline or betaine intake was calculated separately by a food composition database that was established according to the Chinese Food Composition Table^(25)^ and the US Department of Agriculture Food Composition Databases^(24,26)^.

### Assessment of covariates

Data about participants’ social demographics and lifestyles were collected in person by trained interviewers at enrolment using a questionnaire. Pre-pregnancy BMI (kg/m^2^) was calculated by dividing pre-gestational weight (kg) by the square of height (m). The BMI was classified based on the Chinese criteria for adults: underweight BMI (< 18·5 kg/m^2^), normal weight BMI (18·5–23·9 kg/m^2^), overweight BMI (24·0–27·9 kg/m^2^) and obese BMI (≥ 28·0 kg/m^2^)^(27)^. Regarding physical activity, the International Physical Activity Questionnaire^(28,29)^ was applied to estimate participants’ occupational activities and leisure time during pregnancy. The metabolic equivalent (min/week) was computed by using data on frequency and length for each activity. Different activity levels were determined based on the instruction of maternal physical activity assessment during pregnancy^(28–30)^. All the participants were followed till they gave birth. In addition, medical records were reviewed to obtain clinical information (e.g. health history, blood biochemical index examination, gravidity and parity history, delivery mode, pregnancy outcome and complications).

Fasting venous blood was collected with and without EDTA-coated tubes separately from participants during their visit for routine GDM screening. After centrifugation, fasting plasma glucose and serum lipid profile (e.g. TAG, total cholesterol, LDL-cholesterol and HDL-cholesterol) were measured by using a HITACHI 7600-DDP/7600–020 automatic biochemical analyser (Hitachi, Ltd) with the respective commercial assay kits (Biosino Bio-Technology and Science, Inc.). In addition, serum folate and vitamin B_12_ were measured by using the respective commercial assay kit (Abbott Ireland Diagnostic Division, Ireland) according to the manufacturer’s instructions. All the above measurements were conducted by professional staff in a standardised clinical laboratory at the Seventh People’s Hospital in Shanghai. The CV for measurement of TAG, total cholesterol, LDL-cholesterol, HDL-cholesterol, folate and vitamin B_12_ was 1·2 , 1·7 , 1·5 , 1·1 , 2·9 and 2·3 %, respectively.

### Statistical analysis

Categorical variables between cases and controls were summarised as numbers (%) and differences between groups were analysed by the *χ*^2^ test or Fisher’s exact test when cell numbers were less than five. Continuous variables were summarised as mean (s d) or median (IQR) and differences between groups were analysed by Student’s *t* test, Mann–Whitney *U* test or Kruskal–Wallis H-test as appropriate.

Dietary intake of choline or betaine was adjusted for total energy intake by using the residual method^(31)^. Energy-adjusted choline or betaine ingestion was categorised into quartiles based on distributions among controls with the lowest quartile as the reference group. Unconditional logistic regression models were adopted to examine the OR and corresponding 95 % CI of GDM by quartiles of choline or betaine intake. Potential confounders were selected and adjusted in the logistic regression model, which included age, pre-pregnancy BMI, parity, TAG, LDL-cholesterol/HDL-cholesterol and serum folate as appropriate. To test whether the correlations appear linear trend, we employed the median value for each quartile and treated it as a continuous variable in the unconditional logistic regression model.

Additionally, stratified analyses were conducted by using multivariable unconditional logistic regression models to determine whether the aforementioned correlations will be modified by age, pre-pregnancy BMI, parity, TAG and HDL-cholesterol to estimate the multiplicative interactions via including each interaction item. All statistical tests were conducted with R (version 4.1.2). *P* values presented were 2-tailed and *P* values less than 0·05 were considered as significant.

## Results

### Characteristics of the participants

Table 1 summarises the social-demographic and clinical characteristics of 543 participants (168 GDM cases and 375 controls). Compared with the healthy control subjects, women with GDM tended to be older, have higher pre-pregnancy BMI, have more frequencies of gravidity and parity, have higher fasting serum TAG and lower HDL-cholesterol and have higher fasting serum folate.

**Table 1.** Sociodemographic and clinical characteristics among cases and controls among Chinese women in Shanghai, China^*^ (Numbers and percentages; median values and interquartile ranges; mean values and standard deviations)

Variables	GDM		Controls		*P*
	(*n* 168)		(*n* 375)		
	*n*	%	*n*	%	
Age at enrolment, years^†^					
Mean	30·70		28·46		
sd	4·67		4·55		< 0·001
Pre-pregnancy BMI, *n* (%)					< 0·001
< 18·5	13	7·7	55	14·7	
18·5–23·9	82	48·8	228	60·8	
24–27·9	42	25·0	62	16·5	
>= 28	31	18·5	30	8·0	
Occupation, *n* (%)					0·341
No work	57	33·9	115	30·7	
Blue collar	57	33·9	115	30·7	
White collar	54	32·1	145	38·0	
Education, *n* (%)					0·505
Middle school or lower	55	32·7	105	28·0	
High school	43	25·6	96	25·6	
College	67	39·9	160	42·7	
Graduate or above	3	1·8	14	3·7	
Physical activity level, MET-min/week^†^					
Mean	1679·17		1712·70		
sd	791		760·08		0·639
	Median	IQR	Median	IQR	
Total energy intake, kcal/d^‡^	1625·8	1326·21, 1919·03	1584·64	1323·10, 1954·22	0·972
Total cholesterol, mmol/l^‡^					
Mean	5·44		5·62		0·055
sd	1·05		1·01		
TAG, mmol/l^‡^	2·41	1·69, 3·56	2·27	1·80, 2·84	0·008
HDL-cholesterol, mmol/l^‡^	1·77	1·54, 1·99	1·96	1·69, 2·22	< 0·001
LDL-cholesterol, mmol/l^‡^	2·85	2·29, 3·46	3·05	2·51, 3·64	0·084
LDL-cholesterol:HDL-cholesterol ratio^†^	1·68	1·34, 1·99	1·59	1·28, 1·89	0·051
Serum folate, nmol/l^‡^	16·57	11·15, 27·88	12·70	7·00, 16·57	< 0·001
Serum vitamin B_12_, nmol/l^‡^	217·50	158·75, 253·25	221·29	175·50, 229·50	0·477
	*n*	%	*n*	%	
Mode of delivery, *n* (%)					0·546
Caesarean	91	54·2	191	50·9	
Natural	77	45·8	184	49·1	
Gravidity, *n* (%)					0·038
>= 3	67	39·9	124	33·1	
2	53	31·5	101	26·9	
1	48	28·6	150	40·0	
Parity, *n* (%)					0·009
> 1	102	60·7	181	48·3	
0	66	39·3	194	51·7	
Preterm, *n* (%)					0·805
No	161	97·0	360	96·0	
Yes	5	3·0	15	4·0	

GDM, gestational diabetes mellitus; MET, metabolic equivalent.

* Age at enrolment, height and physical activity level were assessed with Student’s *t* test. Total energy intake data were assessed with the Mann–Whitney *U* test. Pre-pregnancy BMI, occupation and education data were assessed with the *χ*^2^ test of independence.

† Data are mean (sd) or *n* (%).

‡ Data are median (interquartile range).

### Association between dietary intake of choline or betaine and gestational diabetes mellitus risk

As shown in Table 2, the energy-adjusted dietary choline consumption was not correlated with GDM risk, after adjusting for age, pre-pregnancy BMI, parity, TAG, LDL-cholesterol/HDL-cholesterol and serum folate. The multivariate OR of GDM comparing the pregnant women in the highest quartile of choline consumption to those in the lowest quartile was 0·77 (95 % CI 0·41, 1·43). Similarly, the energy-adjusted dietary betaine intake demonstrated a null association with incident GDM after controlling for the aforementioned confounders (OR for highest *v*. lowest quartile: 0·80; 95 % CI 0·42, 152) (Table 3).

**Table 2.** OR and 95 % CI of gestational diabetes according to quartiles of energy-adjusted daily choline intake^*^

Variables	Quartile of energy-adjusted dietary choline intake				*P*_for trend_
	Q1	Q2	Q3	Q4	
Total choline, mg/d (median)	307	361	435	605	
Cases/controls, *n*	48/94	39/94	53/93	28/94	
Crude OR	1·00	0·81	1·12	0·58	0·1
95 % CI		0·49, 1·35	0·69, 1·81	0·33, 1·00	
*P*_for coefficient_		0·425	0·6569	0·0533	
Adjusted OR^†^	1·00	0·95	1·27	0·77	0·42
95 % CI		0·53, 1·70	0·73, 2·23	0·41, 1·43	
*P*_for coefficient_		0·8684	0·3982	0·413	

Q1, first quartile; Q2, second quartile; Q3, third quartile; Q4, fourth quartile.

* Unconditional logistic regression was used to estimate OR and 95 % CI.

† Adjusted for age, pre-pregnancy BMI, parity, serum folate, TAG and LDL-cholesterol/HDL-cholesterol.

**Table 3.** OR and 95 % CI of gestational diabetes according to quartiles of energy-adjusted daily betaine intake^*^

Variables	Quartile of energy-adjusted dietary betaine intake				*P*_for trend_
	Q1	Q2	Q3	Q4	
Total betaine, mg/d (median)	53·2	76·3	105	185	
Cases/controls, *n*	37/93	58/94	44/94	29/94	
Crude OR	1·00	1·58	1·19	0·78	0·058
95 % CI		0·96, 2·63	0·71, 2·01	0·44, 1·37	
*P*_for coefficient_		0·0725	0·5156	0·3972	
Adjusted OR^†^	1·00	1·52	1·32	0·80	0·15
95 % CI		0·87, 2·68	0·73, 2·41	0·42, 1·52	
*P*_for coefficient_		0·1463	0·3624	0·5020	

Q1, first quartile; Q2, second quartile; Q3, third quartile; Q4, fourth quartile.

* Unconditional logistic regression was used to estimate OR and 95 % CI.

† Adjusted for age, pre-pregnancy BMI, parity, serum folate, TAG and LDL-cholesterol/HDL-cholesterol.

**Fig. 1.**
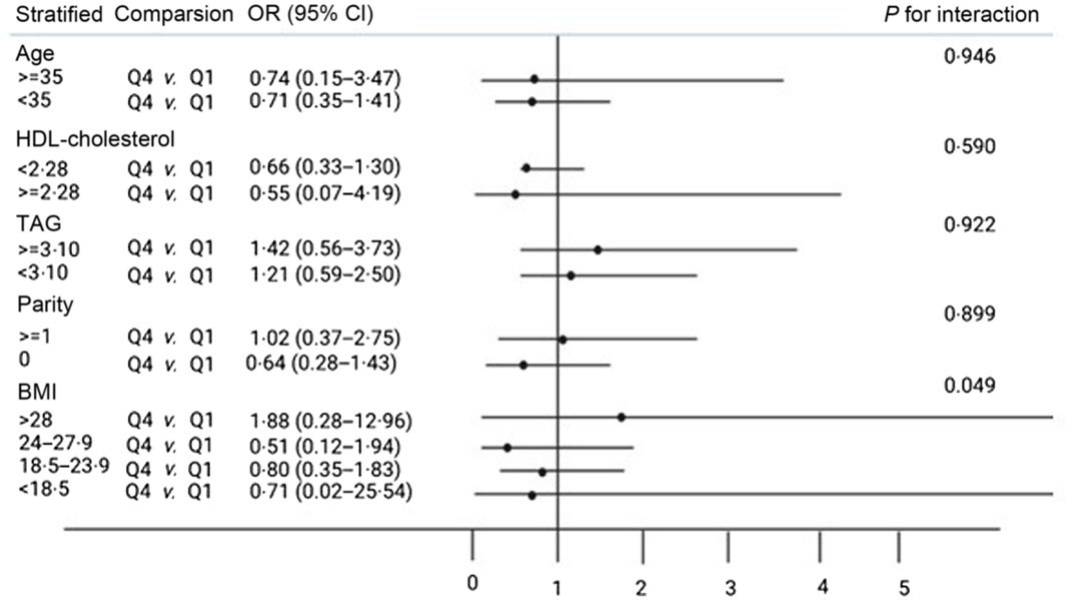
Interaction between the energy-adjusted daily choline intake and risk factors on gestational diabetes mellitus risk. Unconditional logistic regression was used to produce OR and 95 % CI. The model was adjusted for the following cofactors except for the stratification-related factors: age, pre-pregnancy BMI, parity, serum folate, TAG and LDL-cholesterol/HDL-cholesterol. Abbreviations: Q1, first quartile; Q4, fourth quartile.

**Fig. 2.**
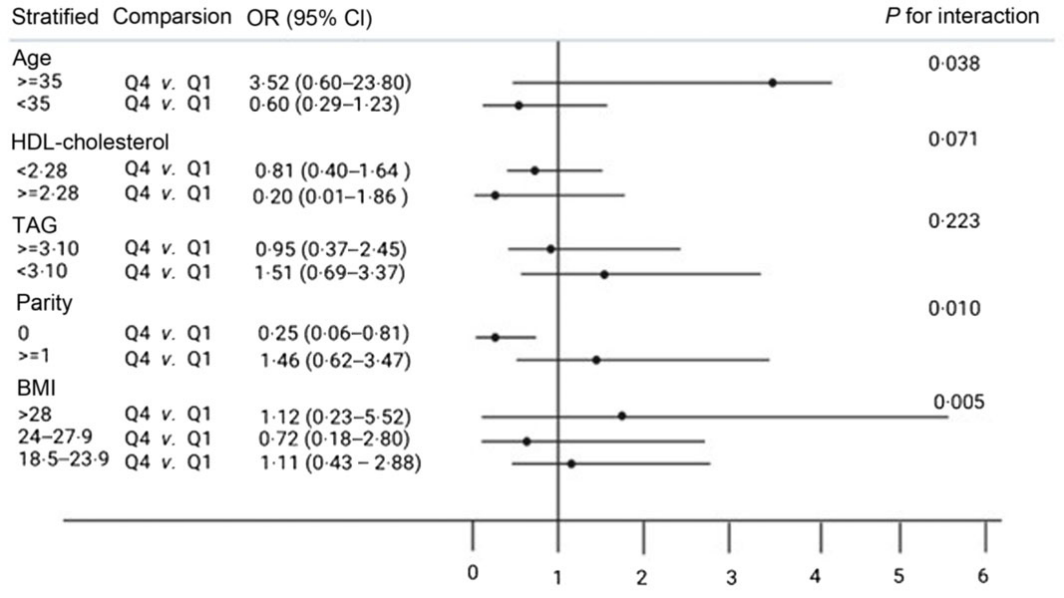
Interaction between the energy-adjusted daily betaine intake and risk factors on gestational diabetes mellitus risk. Unconditional logistic regression was used to produce OR and 95 % CI. The model was adjusted for the following cofactors except for the stratification-related factors: age, pre-pregnancy BMI, parity, serum folate, TAG and LDL-cholesterol/HDL-cholesterol. Abbreviations: Q1, first quartile; Q4, fourth quartile.

Moreover, stratified analyses showed that the null association between dietary consumption of choline or betaine and risk of GDM was not significantly different according to the subgroups of age (≥ 35 years, < 35 years), HDL-cholesterol (< 2·28, ≥ 2·28), TAG (≥ 3·10, < 3·10), parity (≥ 1, < 1) and pre-pregnancy BMI (> 28, 24–27·9, 18·5–23·9, < 18·5) (*P*_for interaction_ = 0·005–0·946) (Figs. 1 and 2). However, there was a significant interaction between betaine intake and parity on the risk of GDM (*P*_for interaction_ = 0·01) (Fig. 2). Among those with no parity history, the multivariate OR of GDM comparing the women in the highest quartile of betaine consumption with those in the lowest quartile was 0·25 (95 % CI 0·06, 0·81) after adjusting the covariates (Fig. 2).

## Discussion

To the best of our knowledge, this hospital-based case–control study is the first to explore the association of dietary choline or betaine intake with the risk of GDM among Chinese pregnant women. We found a null correlation between choline or betaine consumption during pregnancy and GDM risk among the overall participants. However, it is interesting to note that higher betaine intake was related to lower GDM risk among the women who had no parity history before the present pregnancy, and the correlation remained fairly consistent after adjusting multivariable confounders.

It was reported that type 2 diabetes mellitus (T2DM) and GDM shared many common risk factors and pathogenesis^(7,10,17,18,32–38)^. Although previous epidemiology studies investigating the association between intake of choline and risk of GDM were scanty, there were several studies examining the associations between dietary choline intake and incident diabetes. However, the results remain contradictory. In the prospective Women’s Health Initiative cohort, higher consumption of choline was significantly associated with augmented risk of diabetes among 46 263 postmenopausal women for 13·3 years of follow-up^(32)^. Similarly, in the Atherosclerosis Risk in Communities study, choline ingestion was positively associated with T2DM risk among 7392 US middle-aged women over a median follow-up of 9 years^(33)^. A similar unfavourable relationship between choline consumption and diabetes was also observed in a cross-sectional study among 8621 US adult population aged 20 years or older^(34)^. However, a prospective study comprising 2332 males aged 42–60 years in eastern Finland during a mean 19·3-year follow-up reported a negative association between choline consumption and incident T2DM^(35)^. Moreover, another prospective cohort study manifested that dietary choline intake was not associated with T2DM risk with a median 9-year follow-up among 13 440 US participants aged 57–61 years^(33)^. The discrepant results in the above investigations may be partly explained by different ethnicities, diverse frequency of dietary intake measurements and different follow-up periods. In the current study, we observed a null correlation between choline consumption during pregnancy and GDM risk, which warrants more studies to confirm it.

Previous epidemiology studies exploring the association between betaine status and incident diabetes were also limited, and results remained inconsistent. Several prospective cohort studies manifested that betaine consumption was not associated with incident diabetes among postmenopausal US women^(32)^ and in the US middle-aged adults^(33)^. However, other prospective cohort studies reported an inverse association of blood betaine levels with the risk of T2DM among Chinese adults aged 40–75 years^(36)^, Norwegian adults aged 54–70 years^(37)^ and a Dutch cohort of predominantly middle-aged (52·6 (sd 11·5) years) Caucasians^(38)^. Favourable associations of dietary betaine intakes or serum betaine levels with insulin resistance were also found in a cross-sectional study among a general Canadian population^(39,40)^. Moreover, a case–control study that was nested within a prospective cohort of Chinese pregnant women, including 243 GDM cases and 243 controls, manifested that higher serum betaine in early pregnancy was associated with lower GDM risk^(17)^. Likewise, another prospective cohort study reported an inverse association between blood betaine concentration in the second trimester and GDM risk^(18)^. Although we observed the null correlation between dietary betaine consumption during pregnancy and GDM risk among all participants, differences by parity were present. We found that increasing betaine intake was correlated with a lower rate of GDM among the participants with no parity history prior to the present pregnancy. Reasons for these inconsistent findings of epidemiology studies could be attributed to a variety of dietary sources, diversity in participants’ ethnicities and different study designs as well as covariates adopted in the adjustment. Our findings warrant to be confirmed by utilising the biomarkers of dietary betaine intakes.

The specific mechanisms of the role of choline or betaine in the pathophysiology of diabetes are still unclear, although several proposed mechanisms were investigated. Previous studies manifested that both choline and betaine supplementation could enhance cellular insulin sensitivity and improve insulin resistance by suppressing oxidative stress and ameliorating inflammation^(41–44)^. In addition, betaine administration was evidenced to improve gluconeogenesis and glycogen synthesis by enhancing hepatic insulin receptor substrate 1 phosphorylation^(45)^. Betaine could also improve insulin resistance and maintain glucose homeostasis via elevating fibroblast growth factor 21 levels in blood and liver^(46)^ and suppressing the Forkhead box O1 that binds to thioredoxin-interacting proteins^(44)^. Moreover, betaine could participate in the one-carbon unit metabolism via conversion from choline as well as dietary intake and donate the methyl group for modifying the methylation status of genetic loci signals related to insulin signalling^(47)^, which may affect diabetes development. Nevertheless, the above plausible mechanisms warrant further investigation in human studies.

The present study’s strengths include a comprehensive dietary assessment of choline and betaine intakes employing a validated semi-quantitative FFQ, detailed data collection on multiple risk factors as well as potential covariates and using stratified analyses across several GDM risk factors to examine the potentially differential influence of dietary choline and betaine consumptions.

There are also several limitations in the present study. First, because of the nature of the case–control study design, we were unable to infer whether low betaine intake causes incident GDM. Second, residual or unmeasured confounding (such as genetic factors, biomarkers of one-carbon metabolism profile) could not be completely ruled out, although we adjusted for multiple significant potential confounders. Third, although dietary assessment was conducted by a validated semi-quantitative FFQ, the potential recall bias may exist. Furthermore, the participants are Chinese pregnant women living in Shanghai. Owing to genetic variation encoding the key enzymes among diverse populations, various dietary patterns, different living environments and diverse food cultures, the generalisability of our findings may not be applied to other women.

### Conclusions

Although dietary intake of choline during the first and second trimester of pregnancy was not significantly correlated with incident GDM among the Chinese pregnant women, the increasing betaine intake may be related to a lower incident GDM among the Chinese women with no prior parity history. For future research, we suggest conducting studies with a double-blinded, placebo-controlled, randomised clinical trial design to explore the causality and elucidate the underlying mechanisms.
